# Applicabilité de la classification Clavien-Dindo dans l'évaluation des complications postopératoires dans la clinique chirurgicale du Centre Hospitalier National de Nouakchott: analyse observationnelle de 834 cas

**DOI:** 10.11604/pamj.2019.33.254.18024

**Published:** 2019-07-26

**Authors:** Ahmedou Moulaye Idriss, Yahya Tfeil, Jiddou Sidi Baba, Sid'Ahmed Md Boukhary, Mohamed Abdallahi Deddah

**Affiliations:** 1 Département des Spécialités Chirurgicales, Faculté de Médecine, Université de Nouakchott Al Aasriya, Nouakchott, Mauritanie; 2 Centre Hospitalier Régional d'Aleg, Aleg, Mauritanie; 3 Centre Hospitalier National, Nouakchott, Mauritanie

**Keywords:** Complications, postopératoires, classification, Clavien-Dindo, Complications, postoperative, classification, Clavien-Dindo

## Abstract

**Introduction:**

Les complications postopératoires ne sont pas rares; certaines sont transitoires, d'autres peuvent être graves, mais elles sont toutes importantes pour les patients. L'une des plus importantes lacunes dans la recherche chirurgicale est le manque de consensus sur le résultat préféré, comment la mesurer ou l'évaluer.

**Méthodes:**

Nous rapportons une étude rétrospective des complications post opératoires survenues au service de chirurgie générale du centre hospitalier national de Nouakchott. Huit cents trente-quatre patients opérés pendant sept mois (1^er^ janvier 2017 au 31 juillet 2017). Les paramètres démographiques et cliniques ont été étudiés, analysés statistiquement sur le logiciel SPSS 20.

**Résultats:**

Il s'agit de 834 patients dont 426 (51,1%) hommes. L'âge moyen: 34,81 ans (1-90ans). Quatre cents trente-deux (51,2%) patients opérés en urgence. Le sexe ratio (H/F): 1.04. L'ethnie arabo berbère représentait (77,8%). La pathologie appendiculaire (41,12%), pathologie hépatobiliaire (17,76%), pathologie de la paroi abdominale (16,1%). La pathologie thyroïdienne (5,6%). Cent quatre-vingt-trois (21,94%) patients ont développé des complications postopératoires dont 4 (2,1%) décès. Le grade II clavien-Dindo est le plus représenté avec 82,5% des compliquées et représente 17,91% de tous les opérés. L'infection du site opératoire a été de 62.8% de toutes les complications.

**Conclusion:**

Cette étude a montré que la classification de Clavien-Dindo peut être appliquée pour les patients ayant subi une chirurgie élective et une chirurgie d'urgence. Nous pensons que le manque de suivi et le manque de moyen de lutte contre l'infection et le non-respect rigoureux des mesures d'asepsie et d'antisepsie jouerait un rôle important.

## Abstract (en)

**Introduction:**

Postoperative complications are common; some are transient, others may be serious, but they are all important to patients. One of the most important gaps in surgical research is the lack of consensus over the preferred result, the type of measurement or of evaluation.

**Methods:**

We conducted a retrospective study of postoperative complications at the Surgery Department of the National Hospital Center of Nouakchott. Eight hundred thirty-four patients underwent surgery over a seven-month period (1 January 2017-31 July 2017). The demographic and clinical parameters were studied and analyzed statistically using SPSS software 20.

**Results:**

The study involved 834 patients, of whom 426 (51.1%) were men. The average age of patients was 34.81 years (1-90 years). Four hundred thirty-two (51.2%) patients underwent emergency surgery. The sex ratio (M/F) was 1.04. Arab-Berbers race accounted for 77.8%. Appendicular disease accounted for 41.12%, hepatobiliary disease accounted for 17.76%, and abdominal wall disease accounted for 16.1%). Thyroid disease accounted for 5.6%. One hundred eighty-three (21.94%) patients developed postoperative complications, including 4 (2.1%) deaths. Clavien-Dindo grade II was the most represented with 82.5% of complications and accounted for 17.91% of all operated patients. Surgical site infection accounted for 62.8% of all complications.

**Conclusion:**

This study shows that the Clavien-Dindo classification can be applied to patients who have undergone elective surgery and emergency surgery. We think that the lack of follow-up and the lack of means to fight the infection and the non-rigorous respect of asepsis and antisepsis procedures would play an important role.

## Introduction

Après une chirurgie les complications postopératoires (CPO) ne sont pas rares; certaines sont transitoires, d'autres peuvent être graves, mais elles sont toutes importantes pour les patients. La probabilité de complications postopératoires est influencée par le type de chirurgie, l'état préexistant de comorbidité et par la prise en charge péri opératoire des patients [1-3]. Les CPO peuvent être systémiques ou spécifiques aux gestes opératoires et peuvent également être classées selon leur temps d'apparition: immédiat, précoce ou tardif [1, 4]. Il y a près de 20 ans, Richard Horton a décrit quelques lacunes dans la plupart des études chirurgicales, ce qui pourrait être attribué à une mauvaise méthodologie, y compris l'absence de mesures de résultats normalisées [5]. Ces lacunes ont également été identifiées par un groupe interdisciplinaire de chirurgiens, d'épidémiologistes, et de statisticiens qui ont révélé une variété de questions liées à l'évaluation de la chirurgie dans une série de trois articles du Lancet [6, 7]. Ce groupe a indiqué que les plus importantes lacunes dans la recherche chirurgicale étaient le manque de consensus sur le résultat préféré, sa mesure et comment l'évaluer. Une déclaration fiable des résultats est importante pour évaluer et comparer la qualité d'une technique chirurgicale. Des termes tels que des complications majeures, graves ou mineures ont été utilisés d'une manière incohérente, souvent même sans aucune définition. Soucieux de ces lacunes, Clavien et Strasberg ont publié une autre définition des résultats négatifs postopératoires en 1992 [8]. Ils ont proposé un système de classement des complications basé sur le caractère invasif et la thérapie nécessaire pour traiter les complications [8]. Trois types de résultats négatifs après la chirurgie ont été individualisés: les complications post opératoires, l'échec du traitement et les séquelles [9, 10]. Les complications étaient définies comme tout écart par rapport à la normale postopératoire, cette définition prend également en compte les complications asymptomatiques telle que l'arythmie [3]. Une séquelle est un «effet secondaire» de la chirurgie qui est inhérent à la procédure (par exemple, l'incapacité de marcher après une amputation de la jambe), et la chirurgie peut être bien exécutée sans complications. Si le but initial de la chirurgie n'a pas été atteint, cela n'est pas une complication mais un «échec à guérir» (par exemple, une tumeur résiduelle après la chirurgie). Les séquelles et l'échec de guérir ne doivent pas être inclus dans la nouvelle classification de complications [3]. En 2004, une version révisée de ce système a été présentée [9]. Cela était basé sur le même principe, mais excluant quelques critères plus arbitraires, tels que la durée du séjour [9].

Cette classification dite classification de Clavien-Dindo [11] divise les CPO de grade I à grade V, selon le besoin de traitement: Grade I: tout écart par rapport à une évolution postopératoire normale, sans aucun besoin de traitement chirurgical, endoscopique, radiologique ou médical, débridement d'abcès de paroi au lit du malade traitement autorisés: antiémétiques, antipyrétiques, analgésiques, diurétiques, électrolytes et kinésithérapie; Grade II: nécessité de traitements pharmacologiques autres que ceux autorisés ci-dessus; Indication de transfusion ou de nutrition parentérale totale. Grade III: Complication nécessitant un traitement chirurgical, endoscopique ou radiologique: gradeIIIa: sous anesthésie locale, gradeIIIb: sous anesthésie générale; Grade IV: complications menaçantes, y compris neurologiques centrales; indication d'USI (unité de soins intensif): gradeIVa: défaillance d'un organe (y compris dialyse), gradeIVb: défaillance multi-viscérale; Grade V: Décès. L'applicabilité, la simplicité et la variabilité inter-observatrice de ce système de classification révisé a été validée concomitamment dans une grande étude de cohortes [9]. La classification Clavien-Dindo est maintenant largement adoptée et utilisé dans la littérature sur la chirurgie lourde [3, 12]. C'est donc afin d'obtenir des résultats objectifs sur l'activité de notre service de chirurgie générale durant sept mois, que nous avons mené ce travail. Le but de cette étude est d'évaluer le bilan global de notre pratique hospitalière par l'étude de la fréquence des aspects cliniques des complications post opératoires survenues dans le service de chirurgie générale du centre hospitalier de Nouakchott (CHN) pendant une période de sept mois (1^er^ janvier 2017-au 31 juillet 2017) . Dans cette étude nous rapportons l'applicabilité de la Classification de Clavien-Dindo dans la pratique chirurgicale en Mauritanie.

## Méthodes

Étude rétrospective des dossiers des malades opérés en urgence ou en chirurgie élective. Un total de 840 dossiers de patients recueillis sur une période de sept mois (1^er^ janvier au 31 juillet 2017), au service de chirurgie générale d'un centre hospitalier de niveau tertiaire: Centre Hospitalier National (CHN) de Nouakchott en Mauritanie. L'accord du comité d'éthique de l'institution a été obtenu après explication du protocole de recherche. Ont été inclus 834 cas opérés en urgence ou non, hospitalisés dans le service et tous les patients opérés et ayant séjourné au service de réanimation Ont été exclus tous les dossiers incomplets et les dossiers de patients non opérés dans le service. Les caractères démographiques (âge, genre, ethnie), les antécédents médicaux et chirurgicaux ont été étudiés. L'état du risque opératoire a été évalué par le score ASA (American society of anesthesiology). Le diagnostic, le geste le type de chirurgie, la durée d'hospitalisation, le type de complications et le traitement administré ont été analysés. Les complications ont été classées selon les classifications Clavien-Dindo: le grade de complication de chaque patient a été évalué selon la classification de Clavien Dindo. Les grades I et II sont considerés comme complications mineures alors que les grades III, IV et V des complications majeures L'analyse statistique a été réalisée à l'aide du logiciel SPSS 20.0 (Statistical Package for the Social Sciences software for Windows). Les variables utilisées étaient: l'âge, le sexe, l'ethnie, les antécédents, le score ASA, le diagnostic, le geste chirurgical, les complications, le grade de complications, la chirurgie d'urgence, la durée de séjour. Une analyse statistique descriptive a été faite pour évaluer la fréquence, la moyenne la médiane, de chaque variable déterminée. Les variables quantitatives ont été exprimées en moyenne alors que les variables qualitatives ont été exprimées en pourcentage (%). Le risque relatif (RR) de complications en urgence ou non et l'odds ratio (OR) ont été calculés. Une analyse statistique inférentielle a été utilisée et la P-value a été réalisée avec un seuil de signification de P < 0,05. Le CCI (Compréhensives Complications index) a été calculé pour chaque patient par la méthode du calculator CCI^®^ qui est un outil disponible en ligne (*assessurgery.com*) et qui permet l'évaluation en pourcentage des complications postopératoires d'un seul patient ou d'un groupe de patients.

## Résultats

**Figure 1:**
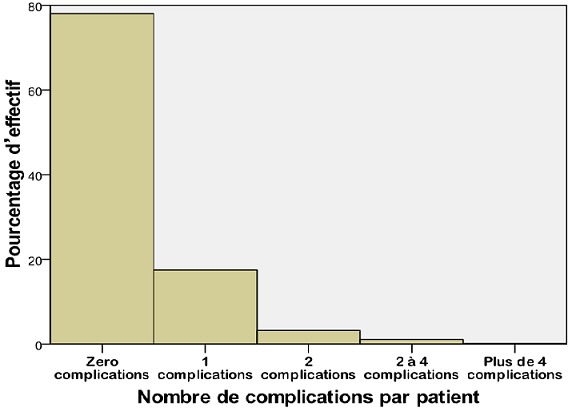
pourcentage d’effectif du nombre de complications par patient

**Figure 2:**
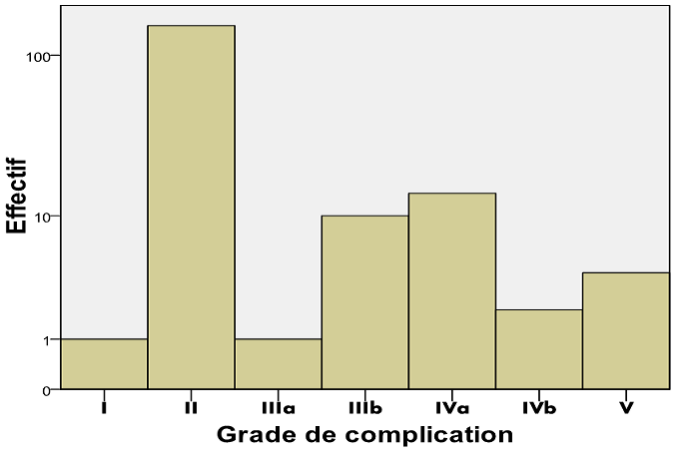
nombre de complications par grade de la classification Clavien-Dindo

**Figure 3:**
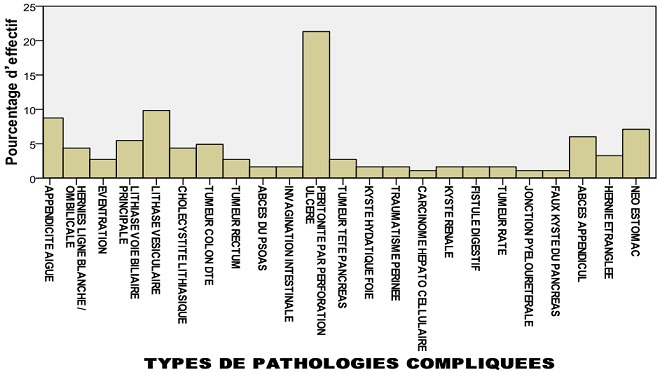
pourcentage d’effectif des pathologies compliquées

Sur 834 patients opérés pendant une période de sept mois, il y avait 426 (51,1%) hommes et 408 (48,9%) femmes. L'âge moyen était de 34,81 ans (extrêmes de 1 à 90 ans) avec 145 (17,38%) patients âgés de 0-16 ans et 98 (11,75%) patients âgés de plus de 60 ans. Le sexe ratio (H/F) était de: 1,04. L'ethnie arabo berbère représentait 651 (77,8%) patients et les non arabo berbères 183 (21,9%) malades opérés. Cent quatre-vingt-trois (21,94%) patients ont développé des complications postopératoires et 651 (78,3%) ont eu des suites simples. Quatre cent trente-deux (51,2%) patients ont été opérés en urgence. Sur les 183 dossiers nous avons pu collecter 245 complications postopératoires, avec 104 (56,8%) hommes, 79 (43,2%) femmes. Le sexe ratio (H/F) des patients compliqués était de: 1,31, leur moyenne d'âge était de: 39,77 ans (extrêmes de 1-90 ans), la médiane de: 40,00 avec 30 (16,39%) patients âgés de 0-16 ans et trente-cinq (19,12%) patients âgés de plus de 60 ans. Pour les patients compliqués l'écart type était de 21,62. L'ethnie arabo-berbère représentait 108 (75,4%) des patients compliqués et les non arabo berbères 45 (24,6%). Parmi les 424 patients opérés en urgence quatre-vingt-quinze (22,40%) ont développé des complications et parmi les 410 opérés à froid 88 (21.46%) ont présenté des complications postopératoires. Trente (16,39%) patients compliqués en postopératoires avaient des antécédents médicaux alors que 49 (26,77%) avaient des antécédents chirurgicaux. Le Tableau 1 présente l'effectif des patients opérés et le pourcentage du genre selon la présence ou non des complications. Odds Ratio (OR) H/F: 0,732 (Intervalle de confiance: 0,525 - 1,019). Pour les cohortes féminins le risque relatif (RR): 1,181 (intervalle de confiance: 0,982-1,420). Pour les cohortes masculins le risque relatif (RR): 0,864 (intervalle de confiance: 0,745-1,002). Le test t de Student a été analysé pour comparer les moyennes et la P-value: 0,064 est supérieure à 0,05, donc il n'ya pas de différence significative entre les 2 groupes du genre. Le pourcentage d'effectif du nombre de complications par patient est représenté dans la Figure 1. Les complications majeures les plus fréquentes pour le CCI sont représentées par les patients ayant un CCI de 20,9 et ceux avec un CCI de 29,6. Le CCI de 20,9 est retrouvé chez 131 (71,5%) patients dont 18 (9,8%) complications majeures et le CCI de 29,6 est retenu chez 17 (9,2%) patients dont 4 (2,1%) complications majeures. La Figure 2 montre que le grade II de la classification Clavien-Dindo est de loin le plus représenté avec un effectif de 151 patients (82,5%) compliquées et 17,91% de toutes les opérées. Le Tableau 2 présente la répartition des principales complications selon le genre avec une nette prédominance des infections du site opératoire en particulier chez les sujets de sexe masculin. Les patients opérés en urgence font plus de complications (Figure 3). L'Annex 1 nous présente quelques exemples de cas de nos patients.

**Tableau 1:** effectif et pourcentage du genre selon la présence ou non des complications

			Genre		
			Masculin	Féminin	Total
Complications	Pas de Complications	Effectif	322	330	652
		%	49,4%	50,6%	100,0%
	Avec Complications	Effectif	104	78	182
		%	57,1%	42,9%	100,0%
	Total	Effectif	426	408	834
		%	51,1%	48,9%	100,0%

**Tableau 2:** effectif et pourcentage du type de complications selon le sexe

Types de complications	Masculin	Féminin	Total
Suppuration de la paroi	71(38.7%)	44(24%)	115(62.8%)
Altération de l’état général	7(3.8%)	5(2.7%)	12(6.5%)
Algies intenses	4(2.1%)	4(2.1%)	8(4.2%)
Anémie	3(1.6%)	4(2.1%)	7((3.7%)
Distension abdominale	3(1.6%)	2(1%)	5(2.6%)
Dysphonie/fausses routes	0	2(1%)	2(1%)
Péritonite post opératoires	1(0.5%)	2(1%)	3(1.5%)
Œdèmes bilatérale des membres inferieurs	2(1%)	1(0.5%)	3(1.5%)
Eventration	0	2(1%)	2(1%)
Rectorragies	1(0.5%)	0	1(0.5%)
Métrorragie	0	1(0.5%)	1(0.5%)
Fistules digestives	0	1(0.5%)	1(0.5%)
Décès	1(0.5%)	3(1.6%)	4(2.1%)

## Discussion

Nous avons réalisés une étude observationnelle rétrospective pour évaluer l'applicabilité de la classification de Clavien-Dindo portant sur les dossiers de malades opérés dans notre service. Cette classification a été appliquée pour les patients ayant subi une chirurgie élective ou une chirurgie d'urgence. Bien qu'il existe un large éventail de mesures de résultats utilisés dans la littérature médicale publiée, il n'existe actuellement aucune définition normalisée des données qui devraient être collectées pour l'appréciation de la qualité d'une chirurgie. Le seul critère d'évaluation utilisé systématiquement en chirurgie est la mortalité [7]. La mortalité pourrait probablement être considérée comme un indicateur raisonnable il y a quelques décennies, alors qu'il y avait encore un risque important de décès après de nombreuses einterventions chirurgicales. Aujourd'hui, cependant, la mortalité est faible même après une chirurgie lourde de sorte que, l'objectif de l'évaluation des résultats s'est tourné vers les complications postopératoires et/ou la morbidité pour évaluer la qualité et les résultats chirurgicaux [13]. Mais une question non résolue est l'absence d'une définition standardisée et l'évaluation des CPO [14-16]. Par exemple, une revue systématique, publiée en 2001, a trouvé plus de 40 définitions différentes des fuites anastomotiques utilisées dans 107 études [17]. L'absence de consensus au sein de la communauté chirurgicale sur la meilleure façon de définir les complications postopératoires a entravé l'évaluation correcte du travail du chirurgien et éventuellement les progrès dans le domaine de la chirurgie. En 1992, Clavien et coll. ont proposé une classification des complications, qui a ensuite été utilisée et révisée par d'autres équipes [8]. La classification de Clavien-Dindo définit la complication chirurgicale comme toute déviation par rapport à l'évolution postopératoire idéale qui n'est pas inhérente à la procédure et ne comprend pas un défaut de guérison. La condition qui reste inchangée après la chirurgie n'est pas une complication mais plutôt un échec de guérir.

Le taux de complications était considéré plus élevé chez les patients opérés en urgence, c'est ce qui ressort dans notre étude. Cependant peu de données de littérature sont disponibles sur les patients opérés en urgence comparativement à celle des malades opérés à froid. Les complications en chirurgie abdominale sont relativement fréquentes [18-20], elles sont liées à la qualité de vie et le coût de leur prise en charge [21, 22]. L'incidence des complications postopératoires diffèrent largement entre les spécialités chirurgicales et même en chirurgie abdominale cette incidence diffère selon le type de chirurgie, alors que la mortalité du fait des progrès scientifiques tend à devenir faible et ne varie que légèrement [21]. En outre, dans la littérature récente 47,5% des patients développent plus d'une complication après une intervention chirurgicale [19]. Même si la classification de Clavien-Dindo a été présentée comme un standard de référence possible par le groupe Balliol [23], le système ne contient pas d'évaluation globale du résultat d'une intervention chirurgicale; souvent, seules les complications les plus graves sont présentées [24]. Pour surmonter ces faiblesses, Clavien-Dindo ont réalisés une série d'études pour améliorer leurs résultats en notifiant des complications postopératoires et offrir une nouvelle évaluation des classifications pour les futures études de recherche sur les complications chirurgicales. Notre travail fait état d'une étude transversale rétrospective présentant la fréquence des complications chirurgicales. Dans cette étude, nous avons examiné si le système de classification Clavien-Dindo largement établi, reflète vraiment la gravité des complications postopératoires en évaluant le nombre, la gravité, et le risque des complications. Nous avons retenu que si tel n'est pas le cas ce système de classification ne serait pas un système utile pour évaluer la morbidité globale après une intervention chirurgicale même si il est largement adopté. Sur la base des résultats de validation du système de classification Clavien-Dindo nous avons cherché à évaluer la morbidité globale de chaque patient par la méthode de calcul du CCI qui n'est autre que le développement et la validation d'un indice de complication complet (CCI) [11]. Ce CCI regroupe toutes les complications postopératoires d'un patient individuel ainsi que leurs différentes sévérités en un seul nombre allant de 0-100 (voir Scenarios des cas cliniques). Un indice de complication aussi complet pourrait devenir la mesure globale des résultats de la morbidité postopératoire [25]. Tous les patients n'ont pas le même risque de complications postopératoires. L'identification des prédicteurs et l'élaboration de scores de prédiction peut aider, avec des mesures préventives efficaces, à réduire le risque pour les complications postopératoires [26-28]. Un score de prédiction pour les complications postopératoires multiples est probablement trop complexe à construire, et de multiples prédictions pour des complications postopératoires spécifiques pourront être considérées pour chaque pathologie. Par conséquent, nous nous sommes concentrés dans cette étude sur le geste opératoire réalisé en urgence en tant qu'issue potentielle de morbidité chez des patients peu ou non préparés, on s'est focalisé aussi sur la précision des facteurs de prédiction.

Dans le cas de notre étude ces facteurs étaient résumés en 3 composantes de facteurs avec la prédominance de l'influence du premier facteur, qui a été étiquetée par: le nombre total de complications, le type de complications, la durée d'hospitalisation et le diagnostic. En dépit de l'inaccessibilité à certaines informations utiles et du caractère rétrospectif de notre étude nous avons fait face à un manque de données locales sur les complications postopératoires en Mauritanie. Il ressort dans notre étude un taux global de complications post opératoires de 21,94% et que parmi les malades compliqués, les infections du site opératoire 115 (62.8%) sont de loin les plus fréquentes avec 77 (42%) hommes. Les décès dans notre série étaient liés à des cancers dans 70% des cas et dans 30% des cas à une défaillance multi- organique, ce qui est comparable au taux rapporté par l'étude rapportée par Mentula *et al*. en Indie [3]. Le taux de patients nécessitant une admission en soins intensif (Grade IV) était similaire à celui rapporté par l'étude réalisé en inde par Mentula *et al*. alors qu'il est inférieur à celui rapporté par l'étude réalisée en suisse par Dindo *et al*.; cela pouvait être expliqué par l'effectif de la chirurgie lourde, l'âge des patients et la différence de taille entre les séries. Le taux de pourcentage: 0,31% des patients ayant subi une ré-intervention sous anesthésie générale (Grade III) est très inférieur aux taux rapportés par les études indiennes: 6,3% et suisse: 4,8%, ceci pourrait être expliqué par le manque de moyens de diagnostic pour les CPO et la proportion élevée des infections du site opératoire (ISO) dans notre étude. Le pourcentage: 17,91% des patients ayant reçu une antibiothérapie (Grade II) en postopératoire est le double de celui rapporté par Mentula *et al*. en inde: 4,2% et il est le triple de celui rapporté par Dindo *et al*.: 7,2%, ce qui pouvait être expliqué par le fait que l'antibioprophylaxie dite de couverture est prescrite systématiquement à tous les patients, plusieurs molécules (seule ou en association) sont prescrites en post opératoire au CHN, cette exagération est très probablement liée aux difficultés rencontrés dans la maitrise des règles d'hygiènes et d'asepsie. Le pourcentage: 0,12% des patients classés en grade I dans notre série est inférieur aux taux rapportés par les études indiennes: 1,6% et suisse: 7,4%, cela pouvait s'expliquer par la proportion élevée d'ISO et l'antibioprophylaxie de couverture systématique au CHN.

L'âge moyen de nos patients est de 34,81 ans, ce qui est proche de celui des séries Maliennes:34 et Camerounaises:38 ans. La moyenne d'âge est inférieure à celle rencontre dans les études rapportées en France et en Inde qui ont rapporté respectivement 54 ans et 50 ans. Cette différence s'explique par la jeunesse de la population au sud du Sahara en général selon l'OMS [29] et en Mauritanie en particulier (d'après le dernier recensement effectué par l'office nationale de statistique (ONS) en RIM 70% de la population mauritanienne est jeune) [30]. La sex-ratio H/F de 1,04 en faveur des hommes pour l'ensemble de la série. Mais ce taux change (pour les malades ayant présenté des complications postopératoires H/F: 1,31), par conséquent les hommes font plus de complications que les femmes dans notre étude. Ce sex-ratio est proche de la série rapportée par Tchalla [1, 3] au Mali [29], contrairement aux études rapportées par Tonye *et al.* [31] et Markus *et al*. [32] qui ont rapportés respectivement un sex-ratio de 1,7 et 1,01. La survenue des complications a augmenté le séjour moyen hospitalier post- opératoire des patients de 4,28 jours à 9,85 jours. Ceci est inférieure à celle rapportée par Tchalla *et al*. (15 jours 23 jours) et Dindo *et al.*: (7 jours 14 jours). Dans notre étude la durée du séjour des patients compliqués a doublé par rapport aux patients non compliqués, et ceci a été rapporté dans la série de Clavien-Dindo. Mais il reste toujours une différence entre les 3 études, liées à la grande variabilité des types d'interventions effectuées dans les services de chirurgie rapportés. La mortalité globale dans notre étude était de 0,48%. Les états de choc (septique, cardiogénique) sont la première cause dans la plupart des études faites en Afrique [33], et la nôtre ne fait pas l'exception. Selon les pathologies la mortalité la plus élevée dans notre étude était liée aux péritonites. Plusieurs facteurs pourraient l'expliquer: notamment le retard de consultation et la mauvaise observance du traitement antibiotique ou encore de leur inefficacité [34]. En ce sens les nouvelles recommandations de la SFAR (Société Française d'Anesthésie Réanimation) pour la prise en charge des infections intra abdominales [35], devraient être plus largement partagées et appliquées dans les différents services de chirurgie abdominale ou mieux encore l'établissement de protocoles d'antibiothérapie adaptés à l'écologie bactérienne de nos services. Les taux de mortalité à titre indicatif sont variés, il ne serait pas opportun de les comparer car les séries diffèrent par leur taille, l'âge des malades, les pathologies, les antécédents, les techniques opératoires etc. En Afrique la plupart des études montraient des taux très élevés de morbidité. Cette morbidité était en grande partie liée à la survenue de suppurations pariétales en post opératoires, ce qui pose le problème des mesures d'asepsie au bloc opératoire et du bon suivi des plaies opératoires dans les salles d'hospitalisation. Notre taux de complications de 21,94% est statiquement supérieur à celui de la série rapporté par Tchalla au Mali (15,4%), par Tonye *et al*. au Cameroun (14,4%) et Dindo *et al.* en Suisse (16,4%), mais comparable à celui des complications rapportées par Mark A *et al*. en Amérique (30.3%), par Mentula en Inde (25,9%) et par Markus *et al*. en Allemagne (29,5%). Cette différence peut être due aux facteurs suivants: le manque de consensus sur la définition d'une complication post opératoire, le manque de moyens pour diagnostiquer les complications, de l'effectif de la chirurgie lourde dans les autres séries. Notre taux d'ISO de 13,64% est supérieur à celui des auteurs des autres pays comme l'atteste les résultats de Tchalla Abalo (Bamako): 6,94%, Tonye TA (Yaounde): 5,28%, Coello R (Angleterre): 4,20% et Mark A, (USA): 6,45%. L'infection étant multifactorielle il est difficile d'expliquer cette différence. Nous pensons que le manque de suivi et le manque de moyen de lutte contre l'infection et le non-respect rigoureux des mesures d'asepsie et d'antisepsie jouerait un rôle important.

## Conclusion

Au vu des résultats de cette étude observationnelle nous concluons qu'après toute chirurgie les complications postopératoires ne sont pas rares et qu'elles sont toutes importantes aussi bien pour le chirurgien que pour le patient. La classification de Clavien-Dindo est l'une des mesures les plus utilisées dans l'évaluation des résultats chirurgicaux publiés dans la littérature médicale. De multiples facteurs interviennent dans la survenu de ces complications. Le manque de suivi des malades, le manque de moyen de lutte contre l'infection et le non-respect rigoureux des mesures d'asepsie et d'antisepsie jouerait un rôle important. L'infection du site opératoire est de loin la complication la plus fréquente. Pour prévenir ces CPO, des recommandations sont nécessaires, à type de conseils, de respect des règles d'hygiène, d'asepsie, de stérilisations des blocs opératoires, de surveillance post opératoire. Cette prévention doit viser d'abord les patients, le personnel médico-chirurgical. Les autorités politiques et sanitaires doivent être aussi impliquées. Enfin cette étude nous a montré que la classification de Clavien-Dindo est applicable dans un contexte africain aussi bien en chirurgie d'urgence ou une chirurgie élective. Les facteurs de prédiction des complications postopératoires dans notre étude sont dominés par le nombre total de complications par patient, le type de complications, la durée d'hospitalisation et la nature du diagnostic.

### État des connaissances actuelles sur le sujet

- complications en chirurgie abdominale sont relativement fréquentes;
- Le seul critère d'évaluation utilisé systématiquement en chirurgie est la mortalité;
- L'incidence des complications postopératoires diffèrent largement entre les spécialités chirurgicales et même en chirurgie abdominale cette incidence diffère selon le type de chirurgie, alors que la mortalité, du fait des progrès scientifiques tend à devenir faible et ne varie que légèrement.

### Contribution de notre étude à la connaissance

- Évaluation pionnière du problème dans notre contexte;
- Les facteurs de prédiction des complications postopératoires dans notre étude sont dominés par le nombre total de complications, le type de complications, la durée d'hospitalisation;
- Cette étude nous a montré que la classification de Clavien-Dindo est bien applicable dans notre contexte africain aussi bien en chirurgie d'urgence qu'en chirurgie élective.

## Conflits d’intérêts

Les auteurs ne déclarent aucun conflit d’intérêts.

## Annexe 1 Dans cette annexe nous présentons quelques exemples de cas de nos patients

**Scénario de cas cliniques:**

**Cas cliniques N 1:**

Patiente de 40 ans aux antécédents d’anémie, classée ASA II et opéré pour tumeur gastrique, chez qui on a effectué une duodéno-pancréatectomie céphalique. Les suites opératoires ont été marquées par des vomissements et un syndrome fébrile.

Diagnostic: suppuration pariétale

Grade: II, CCI: 100

Traitement: tri-antibiothérapie, antipyrétique et transfusion Durée d’hospitalisation : 37jours

**Cas cliniques N 2:**

Patient de 45 ans aux antécédents d’épi gastralgies non étiquetées opéré pour perforation d’ulcère gastrique classaient ASA I .Admis pour hernie inguinale droite chez qui on a effectué une cure d’hernies. Les suites opératoires ont été marquées par des douleurs atroces.

Grade: II, CCI: 51.7

Traitement: bi-antibiothérapie et antalgique Durée d’hospitalisation: 2 jours

**Cas cliniques N 3:**

Patient de 38 ans sans antécédents pathologiques particuliers classé ASA II et admis pour appendicite aigue chez qui on a effectué une appendicectomie. Les suites opératoires ont été marquées par un tableau d’agitation fébrile avec une distension abdominale et un syndrome péritonéal dont l’exploration échographique a mis en évidence une collection intra-péritonéale.

Diagnostic: péritonite postopératoire

Grade: III b, CCI: 20.9

Traitement: ré-intervention et durée d’hospitalisation: 16 jours

**Cas cliniques N 4:**

Patient de 29 ans sans antécédents pathologiques particuliers classé ASA III et opérée pour plaie pénétrante de l’abdomen (brèche du sigmoïde) chez qui on a effectué un élargissement des parois

avec suture. Les suites opératoires ont été marquées par un tableau d’état de choc

Grade: IV b, CCI: 46.2

Traitement: ré-intervention par laparotomie médiane

Durée d’hospitalisation: 13jours dont 3jours en unité de soins intensifs

**Cas cliniques N 5:**

Patiente de 31 ans aux antécédents de thrombophlébite à répétition, classée ASA II, opérée pour occlusion intestinale aigüe sur invagination intestinale aigüe. Elle a bénéficié d’une laparotomie avec résection des anses jejuno-jejunales et rétablissement de continuité termino-terminale. Les suites opératoires ont été marquées par un décès.

Grade: V, CCI: 20.9

Traitement: tri-antibiothérapie, antipyrétique, antispasmodique et dopamine

Durée d’hospitalisation: 61jours dons 6 en unité de soins intensifs
